# An open time-series simulated dataset covering various accidents for nuclear power plants

**DOI:** 10.1038/s41597-022-01879-1

**Published:** 2022-12-13

**Authors:** Ben Qi, Xingyu Xiao, Jingang Liang, Li-chi Cliff Po, Liguo Zhang, Jiejuan Tong

**Affiliations:** 1 Institute of Nuclear and New Energy Technology, Tsinghua University, Beijing, 100084 China; 2 Micro-Simulation Technology, Montville, New Jersey 07045 United States of America

**Keywords:** Nuclear fusion and fission, Power stations

## Abstract

Nuclear energy plays an important role in global energy supply, especially as a key low-carbon source of power. However, safe operation is very critical in nuclear power plants (NPPs). Given the significant impact of human-caused errors on three serious nuclear accidents in history, artificial intelligence (AI) has increasingly been used in assisting operators with regard to making various decisions. In particular, data-driven AI algorithms have been used to identify the presence of accidents and their root causes. However, there is a lack of an open NPP accident dataset for measuring the performance of various algorithms, which is very challenging. This paper presents a first-of-its-kind open dataset created using PCTRAN, a pre-developed and widely used simulator for NPPs. The dataset, namely nuclear power plant accident data (NPPAD), basically covers the common types of accidents in typical pressurised water reactor NPPs, and it contains time-series data on the status or actions of various subsystems, accident types, and severity information. Moreover, the dataset incorporates other simulation data (e.g., radionuclide data) for conducting research beyond accident diagnosis.

## Abstract (summary)

**Table:** 

Measurement(s)	nuclear power plant accident data
Technology Type(s)	nuclear power plant similulation
Factor Type(s)	accident

## Background & Summary

Nuclear energy has been a great discovery in human history. After more than 100 years of development since humans discovered nuclear radiation in the late 19th century, nuclear energy is now closely linked to peoples’ lives and jobs^1^. Nuclear power is one of the main forms through which human beings use nuclear energy to promote economic development and benefit society. Since the first commercial nuclear power plant (NPP) was built in the former Soviet Union in the late 1950s^2^, more than 450 nuclear power units have been in operation worldwide^3^. During this time, in more than 60 years, nuclear power technology has undergone iterative upgrades: from Generation 1 prototype reactors to Generation 2 commercial reactors and then to Generation 3 advanced high-power nuclear reactors. In addition, nowadays, Generation 4 nuclear power systems, which are safer and more economical than the above-mentioned systems, are being explored and experimented^4^. Under the current wave of global industrial intelligence, numerous countries have conducted research on the integration of the technologies of nuclear power and artificial intelligence, especially through the development of digital instrumentation and control (I&C) systems, which can collect large amounts of operational data^5^. However, the value of such large data amounts has not been fully explored, and intelligent nuclear power is still a technique to be developed in the future.

The experience of the previous three serious nuclear accidents in history shows that relying only on nuclear power plant operators to perform early accident diagnosis can result in serious consequences due to human errors^6^. Artificial intelligence (AI) refers to the technology of expressing human-like intelligence through computational models^7^. AI systems can manage complex situations and efficiently process multi-source information, making them suitable for the task of rapidly and accurately diagnosing accidents in nuclear power plants. Therefore, many studies have been performed to develop artificial intelligence-based accident diagnosis technologies for nuclear power plants. Typical AI applications, such as face recognition^8^ and autonomous driving^9^, have been developed by training kernel algorithms (i.e., artificial neural network^10^, support vector machine^11^, decision tree^12^) with massive data with the help of high-performance computing. Algorithms, data, and computing are the three core elements of AI. Among them, the use of datasets has always been a fundamental factor that directly affects the final performance of AI models in real-world scenarios. A high-quality dataset can be a good starting point for validating or building better algorithms. However, regarding nuclear power plants, it is difficult to obtain real accident data, as accidents rarely occur in practice. Moreover, the high safety requirements of NPPs make it impractical to experimentally obtain data from commercial nuclear power plants. Thus, nuclear power plant simulators are often used to obtain large amounts of data.

Nuclear power plant simulation is a technique for simulating system characteristics using mathematical and theoretical models, and it has become an important tool for nuclear power plant design and characterisation. To the best of our knowledge, there are no open datasets for nuclear power plant accident diagnosis. Most studies use non-open nuclear power plant simulators to construct datasets and then train diagnostic models to verify the performance of new algorithms. For example, Yao *et al*. obtained five nuclear power plant accident data using a RELAP5-HD simulator, and they compared the performance of five artificial intelligence algorithms for nuclear power plant accident diagnosis^13^. Qi *et al*. used the simulator of three-loop pressurised water reactor to validate hybrid AI algorithms driven by both knowledge and data^14^. Wang *et al*. used an online pressurised water reactor (PWR) simulator to obtain seven types of nuclear power plant accident data and verify the accident diagnosis performance of a hybrid AI algorithm^15^. Lee *et al*. used another simulator (3KEYMASTER) to obtain ten types of nuclear power plant accident data so as to verify the feasibility of convolutional neural networks for nuclear power plant accident diagnosis^16^. Yang *et al*. studied the capability of various artificial intelligence algorithms for reactor transient analysis based on computational fluid dynamics (CFD) data^17–19^. Moreover, Wang *et al*. developed a simulator called Nuclear Steam Supply System and validated the feasibility of Long Short-term Memory neural networks with regard to small PWR accident diagnosis^20^. In summary, previous studies were usually performed based on private datasets to develop and optimise accident diagnosis algorithms. However, there are several issues with non-open datasets. First, it is difficult to compare the performance of different algorithms due to the lack of a common benchmark dataset. Second, the data quality of the used simulators in some studies may not have been verified with sufficient reliability. Third, the constructed datasets in some studies mostly cannot cover a comprehensive range of accident types. In addition, it is a repetitive effort for researchers to build their own datasets when developing and optimising algorithms.

To address the above-mentioned issues, we built an open dataset, Nuclear Power Plant Accident Data (NPPAD), with massive data that covers various accidents that can occur in nuclear power plants to help with the development and optimisation of artificial intelligence algorithms and other applications. The dataset was constructed based on a nuclear power plant simulation software, PCTRAN, which is one of the most widely used desktop simulators for nearly all types of nuclear reactors. PCTRAN was specifically designed for different light water plant types, such as PWR^21^ and boiling water reactor (BWR)^22^. Since 1998, it has been used by the International Atomic Energy Agency’s (IAEA) annual Advanced NPP Simulation Workshop as a sample model^23^. PCTRAN-based plant-specific models have been installed in nuclear power plants and institutions all over the world for practical applications in training, analysis, probabilistic safety assessment and emergency exercises.

In this study, an open dataset of the most common accidents of PWR nuclear power plants was constructed. The constructed dataset can be used by multiple domains. For example, AI experts can learn about nuclear power plant domain datasets to develop adapted algorithms, while nuclear power experts can use it as a benchmark dataset to compare the performances of various algorithms in NPP accident diagnosis. Notably, in the emerging research area, Gong *et al*. proposed a digital twin technique for nuclear reactor operations, which also presents an urgent need for nuclear power plant accident datasets^24–26^.

In the rest of the paper, we introduce the main NPP structure, theoretical models of PCTRAN, the methods used to generate the proposed dataset, the data records structure, and multiple aspects of technical validations.

## Methods

In this section, we describe the methods used to create NPPAD^27^, as well as a description of nuclear power plants, theoretical models of PCTRAN, and an overview of the processing workflow.

### Description of nuclear power plants

**Fig. 1:**
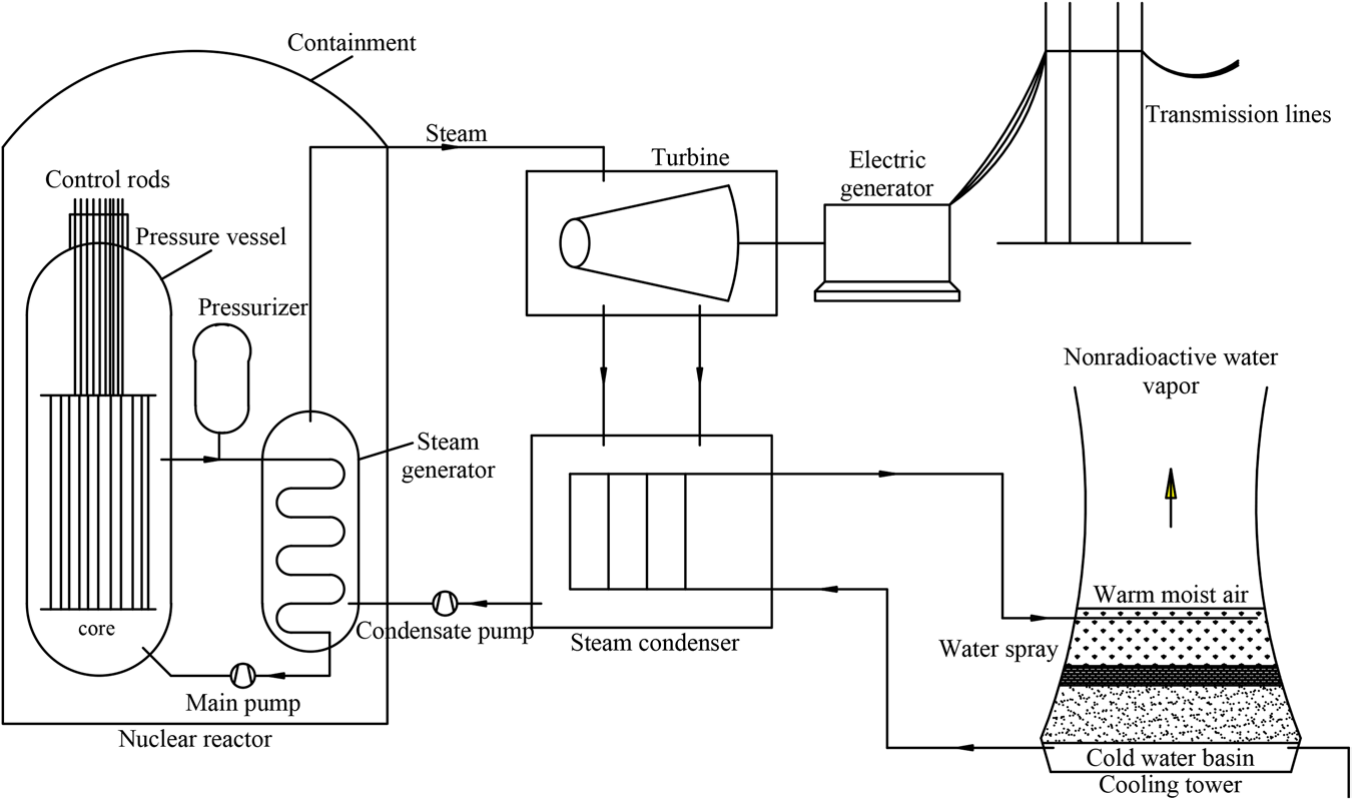
Main structure of a PWR nuclear power plant.

The nuclear power plants currently in operation have numerous reactor types, including PWRs, BWRs and fast reactors, and two-thirds of these reactors are PWRs. Therefore, the constructed dataset in this study is based on PCTRAN. As shown in Fig. 1, the overall structure of a nuclear power plant consists of three main loops. The first loop is the nuclear reactor loop, which consists of a reactor pressure vessel, a pressurizer, a main pump, a steam generator (first loop side), and other components, all of which are located within the containment. The second loop consists of a steam generator, a condensate pump, a turbine, a steam condenser, and other components. The third loop consists of auxiliary system equipment, including electric generators, cooling towers, and other auxiliary equipment. The water in the first loop is heated by the generated heat through nuclear fission and is then transferred to the steam generator, in which it is turned into steam in the second loop. The generated steam rotates the turbine and is then condensed into water, which is returned by a pump to be heated and then turned into steam again. The turbine drives the electric generator, which produces electricity.

### Theoretical models of PCTRAN

**Fig. 2:**
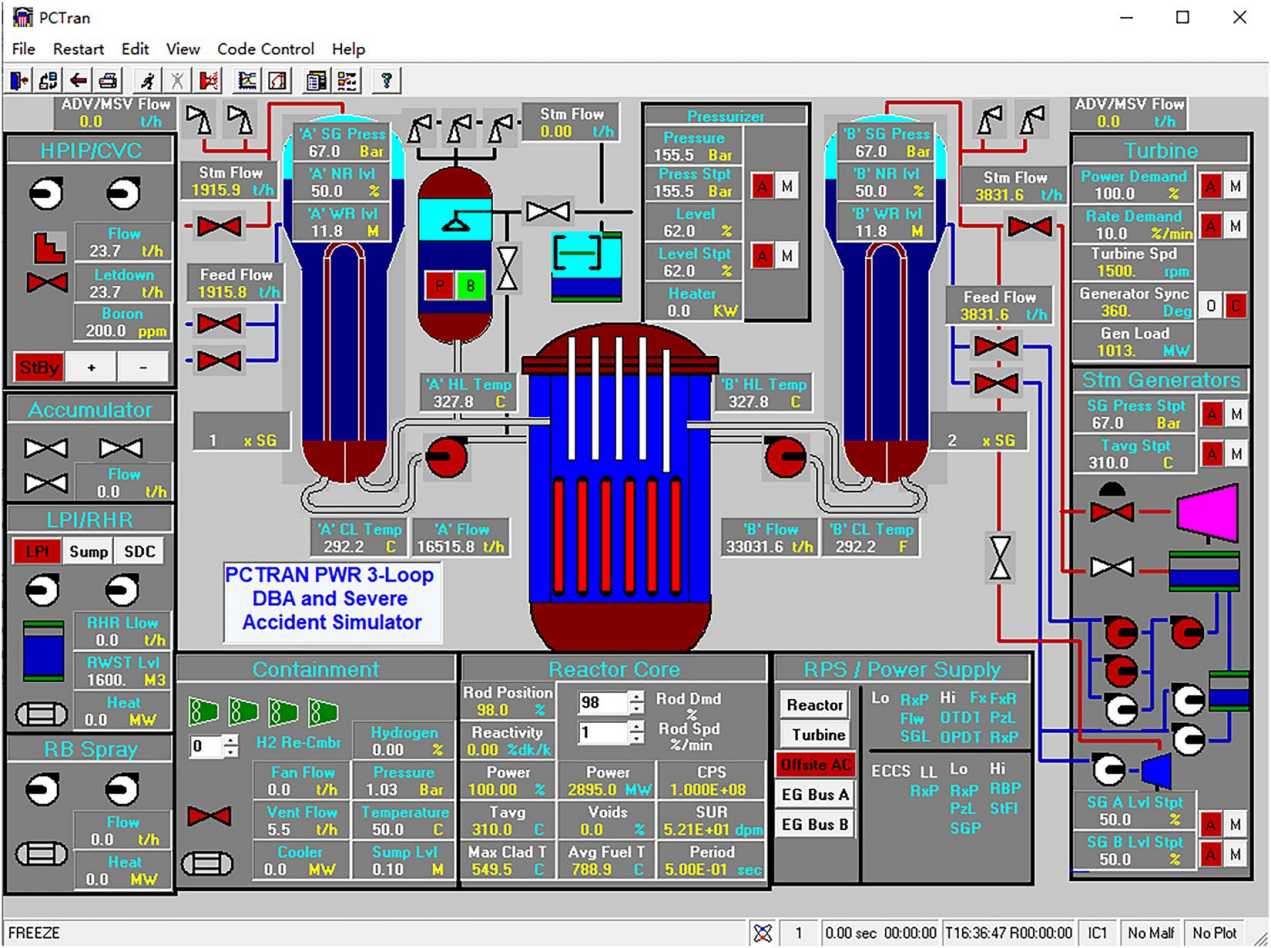
Main control interface of PCTRAN.

**Fig. 3:**
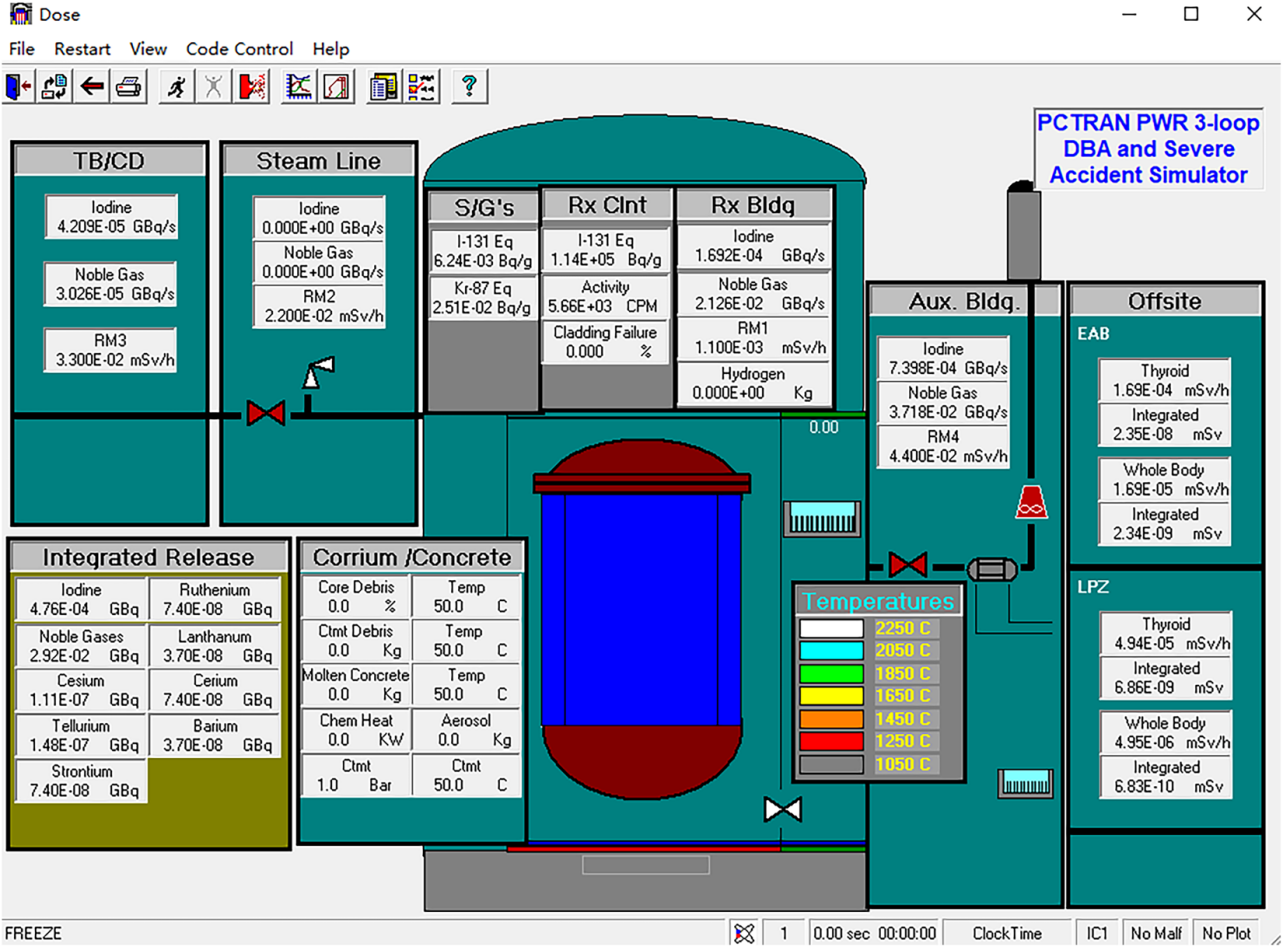
Dose simulation interface of PCTRAN.

PCTRAN is a reactor transient and accident simulation software that is operated based on a personal computer, and it has a Graphical User Interface (GUI) adhering to the specifications of the Microsoft Windows environment. The data input and output are in MS Office’s Access database format (MDB format). PCTRAN provides two control interfaces: the main control interface (Fig. 2) and the radiation dose simulation interface (Fig. 3). In the main control interface, users can control various types of pumps, valves, control rods, and other equipment and can also visualize data on real-time changes in pressure, temperature, flow, and other operating conditions. PCTRAN displays real-time changes in the radiation dose values and cumulative values for each area in the radiation dose simulation interface.

PCTRAN consists of several key simulation modules, including a reactor dynamics module, a reactor coolant system module, and a steam generator module. A brief description of the theoretical models of each module is given below.

#### Reactor dynamics module

This module simulates nuclear reactor cores by describing the variation of neutron densities and related quantities during transients. It includes neutron dynamics^28^, fuel dynamics^29^, steam generator dynamics^30^, and their associated feedback models. As shown in Eqs. 1 and 2, PCTRAN uses a classical set of slow-emitting neutron point reactor models^31^.

1 $$\frac{dn}{dt}=\frac{\rho -\beta }{l}n+\lambda C$$

2 $$\frac{dC}{dt}=\frac{\beta }{l}n-\lambda C$$

where *n* is the neutron density, *ρ* is the reactivity, *β* is the delayed neutron fraction, *t* is the neutron lifetime, *λ* is the decay constant, and *c* is the precursor concentration.

#### Reactor coolant system module

**Fig. 4:**
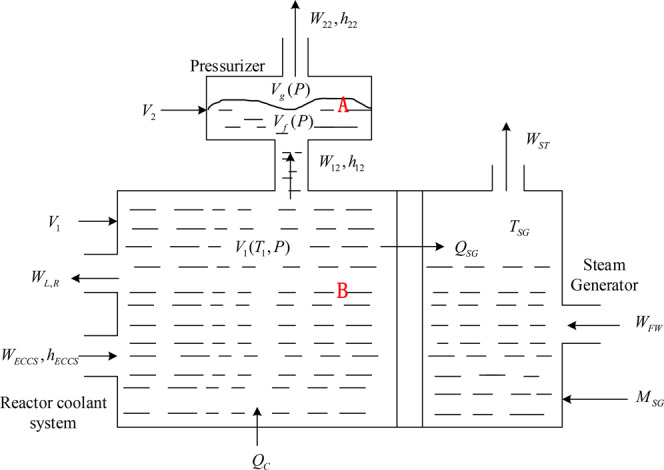
Model of the reactor’s coolant system module and steam generator module in PCTRAN.

This module simulates the reactor coolant system and pressurizer of nuclear reactor loop, and the basic mathematical models are based on the first principles of mass and energy balance, thus ensuring credible and realistic simulations. As shown in Fig. 4, a fluid boundary that separates the saturated two-phase fluid (A) from the subcooled liquid (B) was introduced. The saturated two-phase flow is the fluid within the pressurizer, while the subcooled fluid is the rest of the reactor coolant system’s fluid. In transient operating, the boundary is allowed to move upwards and downwards. The upper two-phase fluid with a total volume *V*_2_ consists of a vapour space, which occupies a fraction *α* of *V*_2_ and a saturated fluid space. The total volume of the lower subcooled fluid is *V*_1_. The A and B fluids are treated separately.

First is the saturated two-phase fluid (A) model^32^. The specific enthalpies and volumes of liquid and vapour are denoted as *h*_*f*_, *h*_*g*_ and *v*_*f*_, *v*_*g*_, respectively. The quality *x* and average mixture enthalpy *h*_*m*_ are related by the following equation^33^:

3 $$x=\frac{\alpha /{v}_{g}}{\alpha /{v}_{g}+(1-\alpha )/{v}_{f}}$$

4 $${h}_{m}=x\cdot {h}_{g}+(1-x){h}_{f}$$

The flow discharge leaving the two-phase volume is denoted by the flow rate *W*_22_ and enthalpy *h*_22_. *W*_12_ and *h*_12_ in Fig. 4 correspondingly express the inter-connecting flow. Then, according to the conservation of mass,

5 $$\frac{d{M}_{2}}{dt}={W}_{12}-{W}_{22}$$

According to the conservation of energy, the nuclear core heat is generated in this volume.

6 $$\frac{dU}{dt}={W}_{12}\cdot {h}_{12}-{W}_{12}\cdot {h}_{22}$$

where *U* is the total internal energy in this volume and is expressed as follows:

7 $$U={M}_{2}\left({h}_{m}-P\cdot {v}_{m}\right)$$

where *M*_2_ is the total mass, *P* is the system pressure and *v*_*m*_ is the average specific volume.

According to the equation of the system’s state,

8 $${V}_{2}={M}_{2}\left[x\cdot {v}_{g}+(1-x){v}_{f}\right]={V}_{2}\left(x,P,{M}_{2}\right)={\rm{constant}}$$

The solution to the system of equations can be obtained by eventually combining the above equations.

Second is the subcooled fluid (B) model^34^. Assuming that the emergency core cooling system (ECCS)’s injection flow is the only flow in this region and that the LOCA break flow *W*_*LR*_ is the net loss, the conservation of mass and energy balance equations in the subcooled region result in

9 $${M}_{1}\frac{d{h}_{s}}{dt}={W}_{EC}\left({h}_{EC}-{h}_{s}\right)-{W}_{LR}\left({h}_{LR}-{h}_{s}\right)-{W}_{12}\left({h}_{12}-{h}_{s}\right)+{V}_{1}\left(\frac{dP}{dt}\right)+{Q}_{C}-{Q}_{SG}$$

where *h*_*s*_, which is the specific enthalpy of the subcooled liquid, is a function of the system pressure *P* and liquid temperature *T*. Similarly, the state equation of the subcooled fluid is expressed as follows:

10 $${V}_{1}={M}_{1}{v}_{{\rm{s}}}({\rm{P}},{\rm{T}})={\rm{constant}}$$

According to the conservation of mass,

11 $$\frac{d{M}_{1}}{dt}={W}_{EC}-{W}_{12}-{W}_{LR}$$

The solution to the equation system can be obtained by eventually combining the above equations.

#### Steam generator module

This module includes the heat flux transfer model, water level dynamic control model and pressure, and steam valve control model^35^. The water level dynamic control model is based on the principle of conservation of mass and energy. According to the feed water flow, steam flows to maintain the dynamic stability of the water level. The heat flux transfer model, i.e., the heat exchange equation between the second loop and first loop, is as follows:

12 $$Q={u}_{w}\cdot {A}_{w}\left({T}_{avg}-{T}_{SG}\right)$$

where *u*_*w*_ is the heat transfer coefficient, *T*_*avg*_ is the average temperature of the reactor’s primary coolant, *T*_*sg*_ is the secondary saturated temperature of the steam generator, and *A*_*w*_ is the wet tube’s surface area.

In addition to the above-mentioned core modules, PCTRAN includes a nuclear fuel model, a containment model, and a radiation dose module^36^, whose radiation dose leakage calculation module is a unique function of PCTRAN.

### Workflow overview

PCTRAN was used in this work to produce accident data on nuclear power plants. However, simulating each accident routinely requires manual key/mouse operations at various steps, such as initialising the operating conditions, selecting the accident type, setting the accident parameters, and ending the accident simulation. Specifically, each accident was inserted at the 20 s moment of full power operation of the nuclear power plant, the time step of data sampling was 10 s and the average simulation time for each accident was ~4000 s. When dealing with a large number of accident scenarios, complex manual operations become very cumbersome and inefficient. In this work, we tried to develop scripts to automate the process and manipulate PCTRAN to generate large amounts of accident data quickly and easily. Using the automation script, a total of 1,217 samples (normal and abnormal operation conditions) were simulated to generate the whole dataset, and the operation took more than 1,350 hours in total with a common desktop computer. Such operations are almost impossible to perform using manual key/mouse operations.

**Fig. 5:**
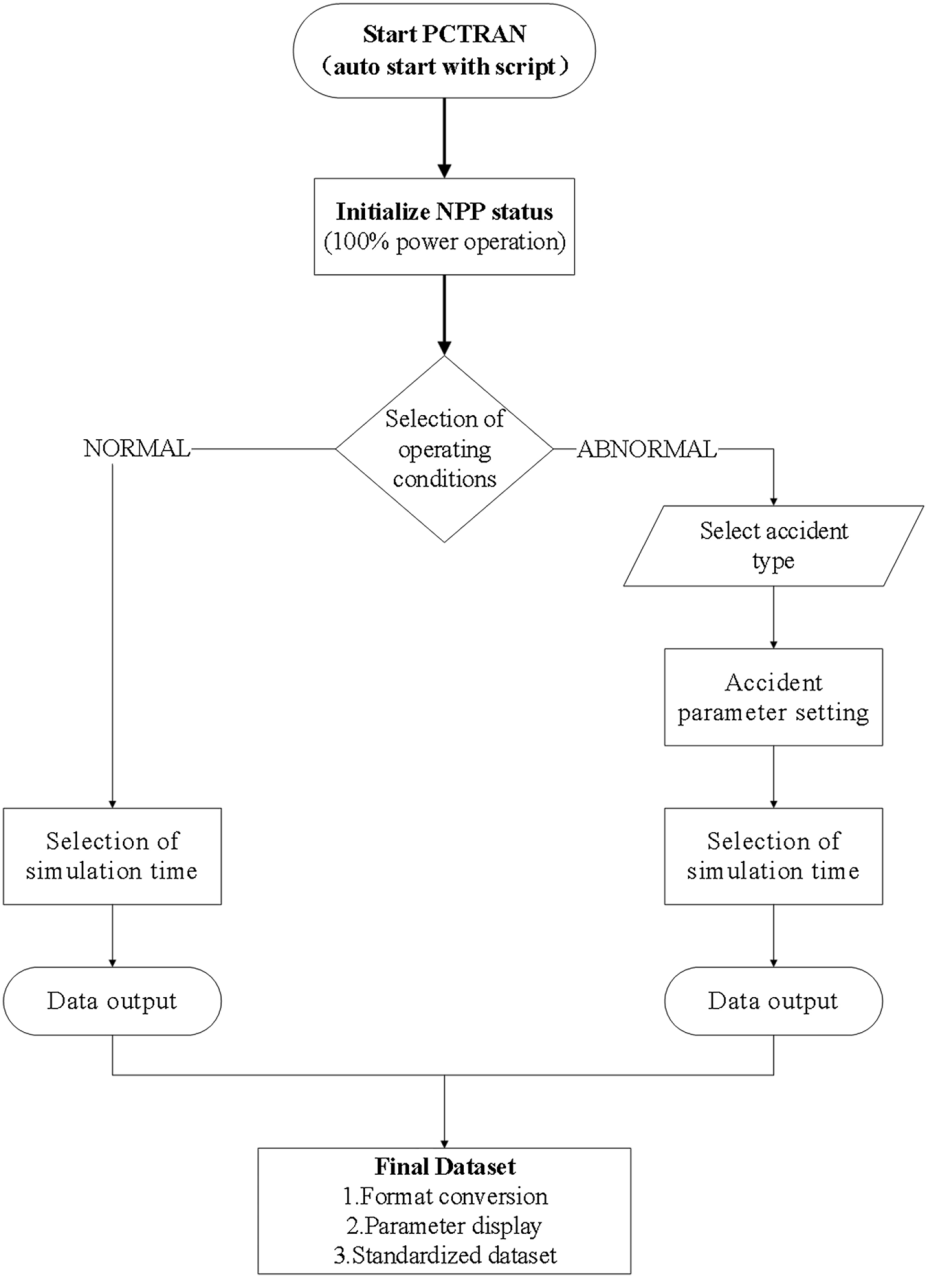
Overall workflow of the simulation data generation.

**Fig. 6:**
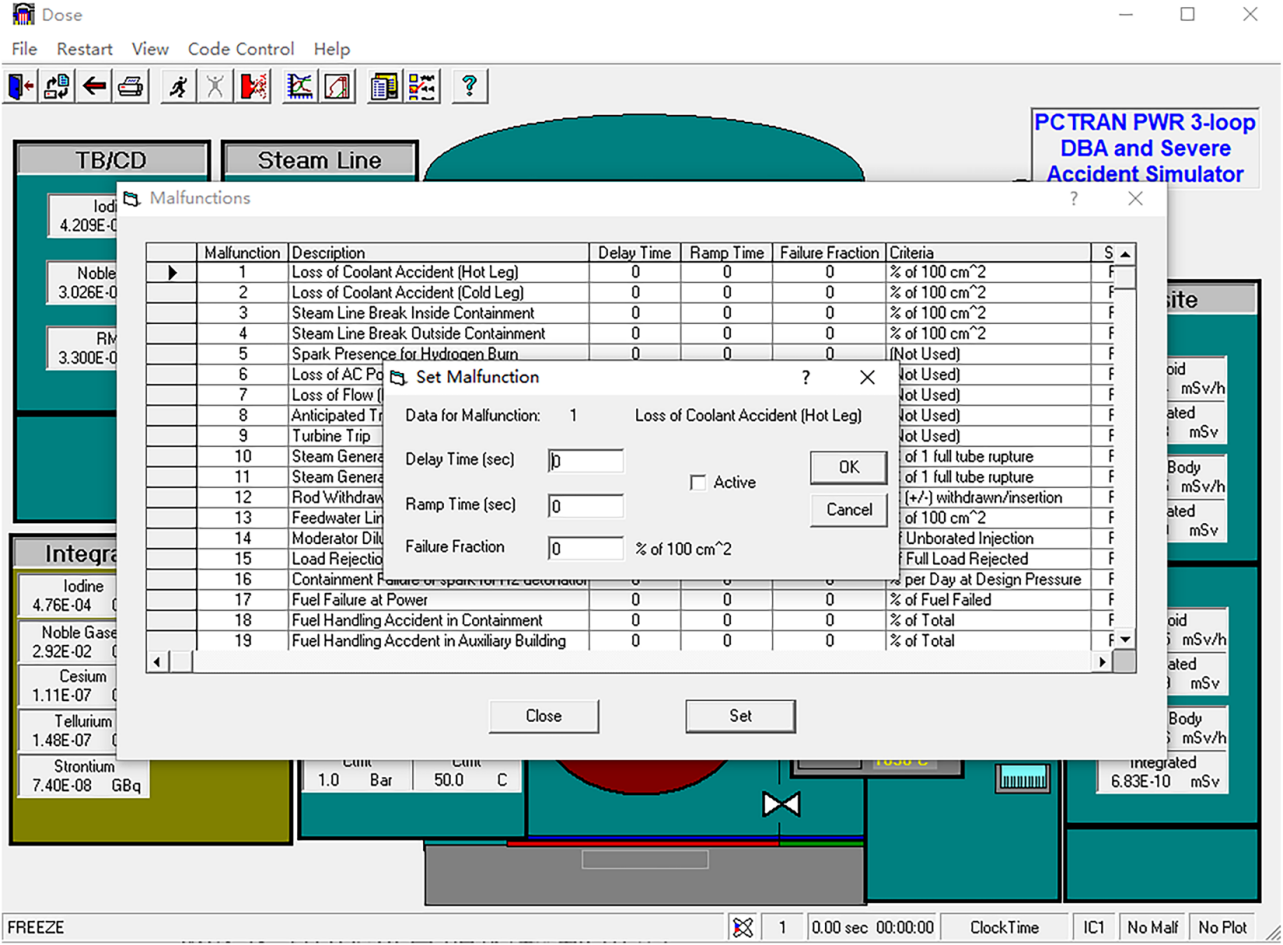
Accident type selection and parameter setting.

The overall workflow implemented in the script to generate the nuclear power plant accident dataset is shown in Fig. 5. First, the PCTRAN software is started by an automation script that replaces the manual key/mouse operations. Once the software is launched, the nuclear power plant (operating at 100% power) is initialised. Then, different operating conditions are selected. If the normal operating condition is treated, the simulator runs for the selected time to get the data output. For abnormal operating conditions (i.e., accidents), as shown in Fig. 6, various parameters, including the accident type, accident parameters, and simulation time, are configured. Then, the simulation data is output. Table 1 lists the accidents covered in this work, where almost all the possible nuclear accidents are simulated. Each of these accidents has the potential to cause reactor core damage, and whether or not they ultimately result in core damage depends on the successful response of the nuclear plant’s accident mitigation system. The dataset in this work does not include cases in which mitigation system failures are superimposed on nuclear plant accidents, as such superimposed cases are too numerous to cover. The detailed process of accident simulation, which is executed by configuring a set of input parameters, is shown in Box 1. If an accident involves different levels of severity, such as the size of the first loop break, it is defined as “severity type,” which needs to be set as a severity parameter. Finally, we obtained the dataset *NPPAD.rar* with normal and abnormal conditions.
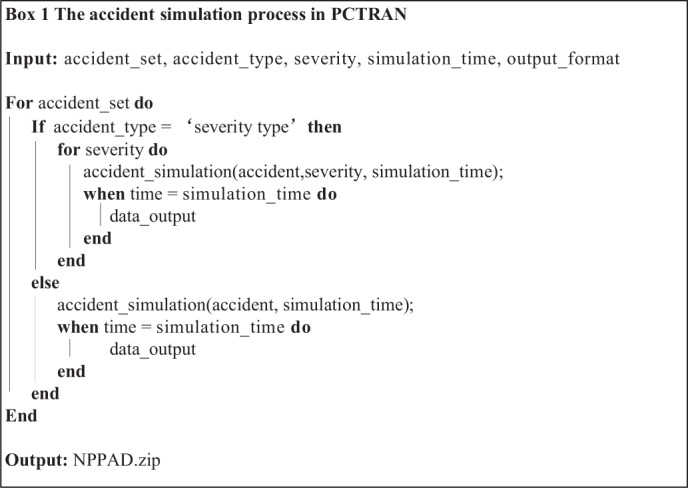

**Table 1:** Accident sets covered by NPPAD.

Accident	Description	Type	Severity
NORM	Normal operating	—	—
LOCA	Loss of Coolant Accident (Hot Leg)	Severity	% of 100 cm^2^
LOCAC	Loss of Coolant Accident (Cold Leg)	Severity	% of 100 cm^2^
SLBIC	Steam Line Break Inside Containment	Severity	% of 100 cm^2^
SLBOC	Steam Line Break Outside Containment	Severity	% of 100 cm^2^
SP	Spark Presence for Hydrogen Burn	Other	—
LACP	Loss of AC Power	Other	—
LOF	Loss of Flow (Locked Rotor)	Other	—
ATWS	Anticipated Transient Without Scram	Other	—
TT	Turbine Trip	Other	—
SGATR	Steam Generator A Tube Rupture	Severity	% of 1 full tube rupture
SGBTR	Steam Generator B Tube Rupture	Severity	% of 1 full tube rupture
RW	Rod Withdrawal	Severity	% (+/−) withdrawn
RI	Rod Insertion	Severity	% (+/−) insertion
FLB	Feedwater Line Break	Severity	% of 100 cm^2^
MD	Moderator Dilution	Severity	% of unborated injection
LR	Load Rejection	Severity	% of full load rejected
LLB	Letdown Line Break in auxiliary buildings	Severity	% of nominal letdown flow

## Data Records

The dataset is available at Figshare^27^. Box 2 illustrates the general structure of the data records in NPPAD, in which accidents are stored in separate directories. The initial version of the dataset contains 18 types of operating conditions that are possible under the full power operation of a three-loop pressurised water reactor NPP. Each operating condition sample contains three files: two in the MDB format and the other in the plain text format. The MDB files can be opened directly using Microsoft Access. For example, as shown in Box 3, the *1.mdb (PlotData)* represents the time series of the status parameters with a 1% of 100 cm^2^ break of LOCA. Moreover, *PlotData* represents the sub-table in the *1.mdb* file. As shown in Box 6, another useful sub-table is *ListPlotVariables*, as it describes the parameters corresponding to the abbreviations in *PlotData*. As shown in Box 4, *1Dose.mdb* represents the time series of the radionuclide in the nuclear power plant. In addition to the MDB format, we also provided a CSV format in the folders *Operation_csv_data* and *Dose_csv_data*. As shown in Box 5, *1Transient Report.txt* describes the actions in the subsystems of the nuclear plant over the simulation time for each accident, which can help users understand changes in the plant status. The numbers in front of the files in the other operating conditions (e.g., *1.mdb*, *2.mdb*) correspond to the severity of the accident, and the exact meaning can be determined by the column ‘severity’ of Table 1. The above-mentioned datasets are also stored on the GitHub website (https://github.com/thu-inet/NuclearPowerPlantAccidentData).
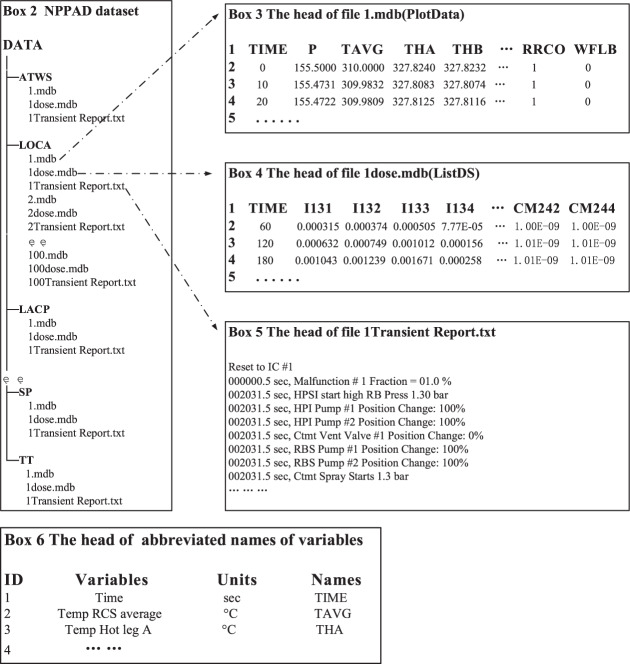


## Technical Validation

Since its introduction in 1985, PCTRAN has been constantly upgraded and expanded. The current software’s scope covers numerous types of PWR and BWR plant designs, including both Generation II and Generation III plants. PCTRAN models have generally gone through detailed benchmarking and verifications. For example, in the International Atomic Energy Agency (IAEA)’s handbook^37^, PCTRAN was used to simulate the nuclear accident of Three Mile Island (TMI), which happened at 4 am on March 28, 1979, when the reactor was operating at 97% power. The accident occurred due to a relatively minor malfunction in the secondary cooling circuit, which caused the primary coolant’s temperature to increase. In turn, the reactor was automatically shut down, which took approximately one second. At this point, a relief valve failed to close; however, instrumentation did not reveal this fact. A large amount of the primary coolant was then drained so that the residual decay heat in the reactor core was not removed. Thus, the core was severely damaged. The TMI accident simulation was analysed for up to 6,000 seconds, where the changes in key parameters, such as the water level of the steam generator, reactor coolant temperature and pressure, and fuel temperature, were accurately presented.

In this section, two simulations were conducted to demonstrate the accuracy and reliability of the PCTRAN simulation software with regard to nuclear power plant operations. The first simulation involved simulating the evolution of the Fukushima nuclear accident, and the simulation results were compared with the measured results (accident report data). The second simulation involved simulating two nuclear power plant operating conditions (load rejection and Large LOCA), and an analysis was performed to see whether the simulation results conformed to the expected physical phenomena.

### Validation using the Fukushima nuclear accident

The PCTRAN simulation was validated against the accident progression of the Fukushima Daiichi nuclear power plant Unit 1, as shown in Table 2^38,39^. As a result of the tsunami, all the cooling capability was lost In Unit 1, which fell into a severe condition within 3 or 4 hours after the earthquake. It was not until the next morning (March 12) that Tokyo Electric Power Company (TEPCO) could inject water into RPV. Then, Primary Containment Vessel (PCV) venting was conducted at 14:30 on March 12. Afterwards, a hydrogen explosion occurred.

**Table 2:** Chronological accident description for Unit 1 of the Fukushima Daiichi NPP.

Date	Time	Event
2011/3/11	14:46	Earthquake: reactor was automatically shut down. Decay heat was continuously generated
		Loss of off-site power: diesel generators were automatically started. Therefore, AC and DC power was available in this period
	14:52–15:34	IC cooling: reactor was cooled by Isolation Condenser (IC) with start-stop operation so that RPV cooling down rate did not exceed 55 °C/h. Unit 1 was operated to achieve a cold shutdown
	15:37	Tsunami hit: AC and DC were lost. IC was not in operation at this time
	After tsunami	Reactor Pressure Vessel (RPV) water inventory decreases due to no water injection
	After 20:00	(PCV) pressure increased
		RPV bottom damage: Corium (melted fuel) slumping to PCV pedestal
2011/3/12	14:30	Regarding the containment vessel vent, the operation of AO valve of suppression chamber side was implemented at 10:17 am, and a pressure decrease was confirmed at 2:30 pm
	15:36	Reactor building explosions

**Fig. 7:**
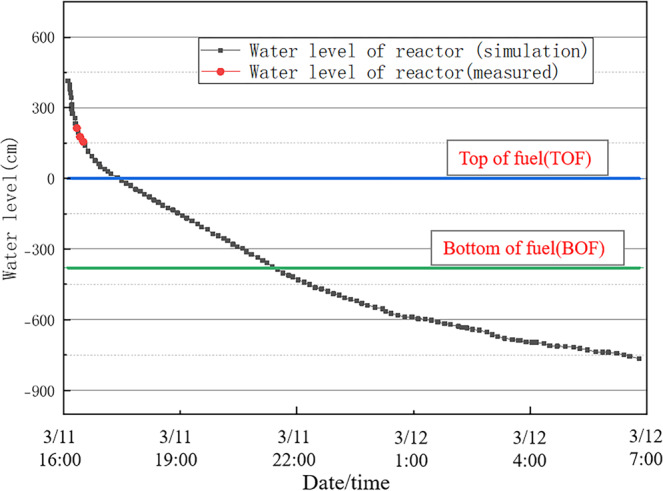
Reactor water level for Unit 1 of the Fukushima Daiichi NPP.

**Fig. 8:**
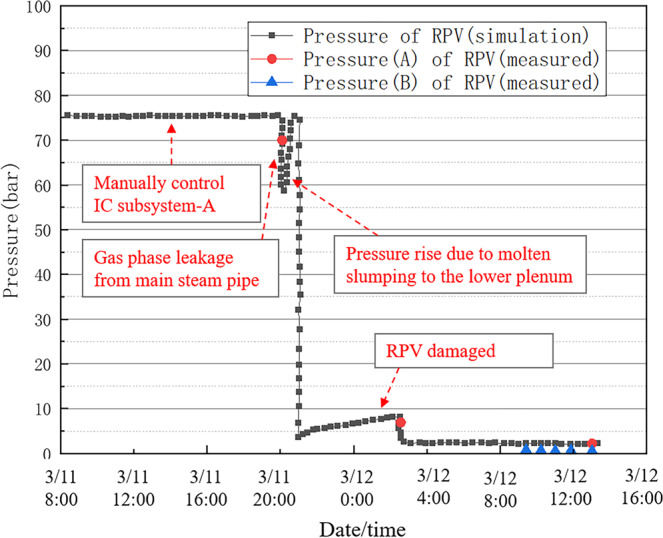
RPV pressure changes for Unit 1 of the Fukushima Daiichi NPP.

**Fig. 9:**
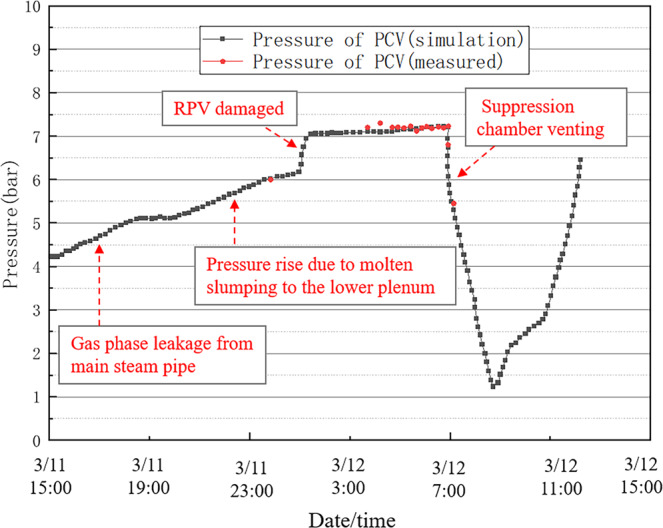
PCV pressure changes for Unit 1 of the Fukushima Daiichi NPP.

The simulation of the accident was performed using PCTRAN with assumed boundary conditions starting with the loss of off-site power, which was induced by the earthquake and tsunami, followed by the venting of the over-pressurised containment unit and the later injection of seawater^40^. The results are shown in Figs. 7–9. Figure 7 shows the change in the reactor water level, while Figs. 8,9 show the changes in the pressures of the reactor pressure vessel (RPV) and primary containment vessel (PCV). The real accident progressions are marked in Figs. 7–9. It can be noted that only limited measurements could be used due to the damage to the monitor from the accident^41^. In this study, for convenience purposes, we divided the accident process into four intervals, and the simulation results were compared with the measured data in each interval.

#### From the Earthquake to tsunami arrival

The reactor pressure increased due to the earthquake-caused shut down, and two isolation condenser (IC) systems were automatically activated. Afterwards, the two IC systems were manually shut down, and then an IC Subsystem-A was activated. As shown in Fig. 8, the reactor pressure was controlled by manually starting up and shutting down the IC subsystem-A to keep the pressure at a certain level.

#### From the tsunami arrival to reactor water level decrease

All the cooling capabilities, including the steam-driven cooling system and motor-operated pump, were lost due to the loss of control power. The water in the reactor continued to boil and evaporate, causing the reactor water level to continuously decrease (Fig. 7). From approximately 16:42 to 17:00 on March 11, 2011, the reactor water level could be measured for some time due to the temporary recovery of some DC power. As observed at 16:56, the water level was at the top of fuel (TOF) + 2,13 cm and had not yet decreased to TOF.

#### From the reactor water level decrease to pcv pressure increase

The RPV pressure was measured as 70 bar at 20:07 on March 11 and as 9 bar at 02:45 on March 12 (Fig. 8). The PCV pressure was measured as 6 bar at ~23:50 on March 11 (Fig. 9). It was observed that at a certain time after 20:00 on March 11, the RPV pressure decreased despite, and the PCV pressure showed a sharp increase, which is considered to be due to gas leakage from the main steam pipe.

#### From the containment vessel pressure increase to containment venting operation

On March 11, at ~23:50, the PCV pressure was 6 bar. However, it then increased and remained near 7–8 bar until the suppression chamber was successfully vented (Fig. 9). The reason was that the molten fuel descended to the bottom of the reactor vessel. Then, it further descended to the bottom of the PCV, thus further increasing the PCV pressure. When the molten fuel could not be sufficiently cooled, the concrete of the PCV floor was heated above its melting point, and a core–concrete reaction started, producing non-condensable gases, such as hydrogen and carbon monoxide, which have dramatic effects on the containment pressure.

For all the intervals, the simulation results correctly showed the control logic and transient processes of Unit 1 of Fukushima Daiichi NPP. Partial measured data, which was acquired for using the temporary recovery of the power supply, was compared with the simulation results. Figures 7–9 show that the simulation results match the measured data. Thus, the reliability and quality of the used PCTRAN data were validated.

### Validation using two operating conditions

Two representative accident conditions of nuclear power plants were selected for the further technical validation of PCTRAN.

#### Normal operation with load rejection

**Fig. 10:**
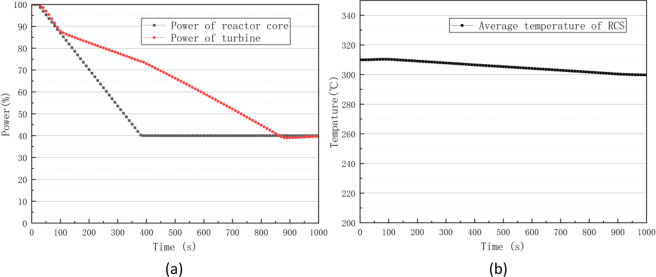
Power curves of reactor core and turbine (**a**) and average temperature curve of RCS (**b**) in a load rejection accident.

Failures in the speed control system of turbines or misclosures of steam piping valves can cause load rejection, and such conditions are among the common accident in nuclear power plants. Through PCTRAN simulations, the processes of reactor core power and turbine power (load) changed after inserting load rejection accidents at 20 s, as shown in Fig. 10a, where the turbine power first changed. Then, the turbine power reduction caused a heat imbalance in the second loop, in turn causing an increase in the average temperature of the first loop. As shown in Fig. 10b, the negative feedback effect of the temperature caused the core power to start decreasing.

**Fig. 11:**
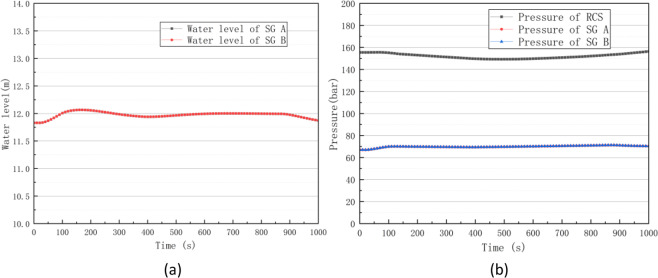
Pressure curves of the RCS and steam generator (**a**) and water level curve of steam generator (**b**) in a load rejection accident.

The first loop pressure increased with the increase in temperature. Then, both of them began to decrease when the spray system and control rod drive system started working. As shown in Fig. 11a, the turbine power decrease also caused a brief pressure increase in the second loop. The pressure gradually decreased as steam was released to the outside through the atmospheric bypass valve. As shown in Fig. 11b, the water level of the steam generator (SG) temporarily increased due to the steam flow reduction. Afterwards, it gradually returned to normal through the control system. During this time, an oscillatory behaviour was caused by the pressure relief of the atmospheric bypass valve.

The above simulation results accurately demonstrate the control logic and transient processes of the load rejection accident.

#### Normal operation with a large LOCA

**Fig. 12:**
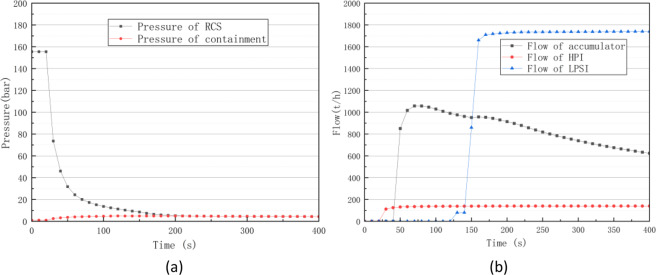
Pressure curves of the RCS and containment (**a**) and flow curve of auxiliary system (**b**) in a large LOCA accident.

Coolant losses due to coolant pipe breaks are also among the common accidents in nuclear power plants. Under full power operation, a large break with an area of 2,300 cm^2^ was assumed. As shown in Fig. 12a, a large coolant amount was discharged, and the reactor’s cooling system pressure rapidly decreased. The containment pressure rapidly increased after the accident and gradually decreased as the containment spray system was operated.

**Fig. 13:**
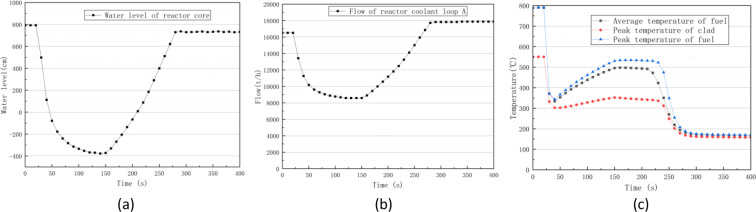
Water level curve of the reactor core(**a**), flow curve of RCS(**b**) and temperature curves of reactor core in a Large LOCA accident.

As shown in Fig. 12b, the high-pressure injection (HPI) system, accumulator, and low-pressure safety injection (LPSI) system were successively put into operation as the pressure of the first loop was decreased to each threshold value. Figure 13a,b show that the core went through uncovered and re-flooded phases, with the core water level first decreasing and then gradually increasing. As shown in Fig. 13c, the fuel clad and peak fuel temperatures significantly increased during the core uncover phase and then decreased again as the core was re-flooded.

The above simulation results correctly show the control logic and transient response of a large LOCA accident.

## Usage Notes

The dataset is in the original MDB format and has a total size of ~15.1 GB. A more detailed description of the dataset and Python scripts for exploring the dataset are available on the GitHub page of the dataset (https://github.com/thu-inet/NuclearPowerPlantAccidentData). Users can reproduce the dataset using the PCTRAN software and automation scripts described in this work. However, it is recommended to directly use the pre-built datasets as the building process is quite time-consuming. The *Data Processing.py* file provides the python code for converting the dataset’s MDB format to Excel. Users can also use our code to generate datasets needed for AI models (e.g., training and test sets). In addition, the code can be used to plot time series graphs.

## Data Availability

These simulations were conducted using PCTRAN-PWR3LP (https://github.com/thu-inet/NuclearPowerPlantAccidentData/tree/main/Simulator/). The data processing step was performed using scripts written in the Python 3.10 programming language. More about this dataset can be found on the dataset’s GitHub page (https://github.com/thu-inet/NuclearPowerPlantAccidentData).
